# Evolution by leaps: gene duplication in bacteria

**DOI:** 10.1186/1745-6150-4-46

**Published:** 2009-11-23

**Authors:** Margrethe H Serres, Alastair RW Kerr, Thomas J McCormack, Monica Riley

**Affiliations:** 1 Josephine Bay Paul Center, Marine Biological Laboratory, Woods Hole, MA 02543, USA; 2 Wellcome Trust Centre for Cell Biology, University of Edinburgh, Kings Buildings, Mayfield Road, Edinburgh EH9 3JR, UK; 3 Laboratory of Neurobiology, National Institute of Environmental Health Sciences, National Institutes of Health, Department of Health and Human Services, Research Triangle Park, NC 27709, USA; 4 Josephine Bay Paul Center, Marine Biological Laboratory, Woods Hole, MA 02543, USA

## Abstract

**Background:**

Sequence related families of genes and proteins are common in bacterial genomes. In Escherichia coli they constitute over half of the genome. The presence of families and superfamilies of proteins suggest a history of gene duplication and divergence during evolution. Genome encoded protein families, their size and functional composition, reflect metabolic potentials of the organisms they are found in. Comparing protein families of different organisms give insight into functional differences and similarities.

**Results:**

Equivalent enzyme families with metabolic functions were selected from the genomes of four experimentally characterized bacteria belonging to separate genera. Both similarities and differences were detected in the protein family memberships, with more similarities being detected among the more closely related organisms. Protein family memberships reflected known metabolic characteristics of the organisms. Differences in divergence of functionally characterized enzyme family members accounted for characteristics of taxa known to differ in those biochemical properties and capabilities. While some members of the gene families will have been acquired by lateral exchange and other former family members will have been lost over time, duplication and divergence of genes and functions appear to have been a significant contributor to the functional diversity of today’s microbes.

**Conclusions:**

Protein families seem likely to have arisen during evolution by gene duplication and divergence where the gene copies that have been retained are the variants that have led to distinct bacterial physiologies and taxa. Thus divergence of the duplicate enzymes has been a major process in the generation of different kinds of bacteria.

**Reviewers:**

This article was reviewed by Drs. Iyer Aravind, Ardcady Mushegian, and Pierre Pontarotti.

## Background

When Charles Darwin wrote *The Origin of Species*, no data existed that could inform him of the molecular nature of genetic variation that fuels evolutionary change. Today the existence of sequences of entire genomes and the ability to compare related sequences allows identification and characterization of sources of genetic variation. Evolution at the molecular level is now known to have taken place through both selection and neutral drift acting on genetic variation arising from many avenues: single base changes, horizontal transfer of genes, loss of genes, rearrangements of genomic segments and, discussed here, gene duplication followed by divergence of the copies. The comparative analysis of sequences of related and unrelated bacteria has filled out our understanding of some of these mechanisms of evolution.

Views of the nature of genetic change underlying evolution have changed over the last century. Koonin has summarized the history of these changes up to the present view [1]. In the beginning, Darwin thought that genetic changes were small and evolution was gradual. This view was maintained as plausible after the structure of DNA became known. Successive single nucleotide changes by point mutation would be small, conforming to the view of the gradual nature of the process. Evolutionary change according to this gradualist view was brought about by selection, that is the fixation of beneficial mutations, elimination of the deleterious. Subsequently Kimura [2] and others introduced the neutral theory, stating that selectively neutral mutations dominate and fixation occurs by random drift. At this time, the type of genetic change was still viewed as gradual accumulation of point mutations.

However in 1970, Ohno [3] introduced the idea of gene duplication as an important form of genetic variation, a process that would go beyond gradualism and would permit quantum changes. The process of gene duplication in microbes as the agent of evolution of novel gene functions is being studied by many scientific groups today e.g. [4-7]. Another source of sudden change was the discovery of horizontal transfer of genes from one organism to another not necessarily related organism [8]. Both these mechanisms, gene duplication and lateral transfer, have the capacity to bring about relatively large changes.

With availability of complete genome sequences of many bacteria, studies have used such data to understand the power law behavior of sizes of paralogous groups of genes in many bacterial species [4]. Others have used collections of genomic sequence data to enumerate types of fates of ancestral genes, concluding that there has been a great deal of loss following duplication, that selection for novel functions has played a prominent role and that rates of divergence of paralogous genes depends on selection pressure and functional constraints [6]. Gevers et al. [7] analyzed presence of sequence-related groups from a functional standpoint. They found that in all the genomes, largest families contained transport genes and regulation genes, smaller families were involved in metabolism and energy production. They considered that duplicated genes were retained if adapted to a changing environment.

As distinguished from such studies of sequence-related families in large data sets like collections of whole genome sequences, we planned to examine a few paralogous groups in a limited number of bacteria where the great majority of the functions of the individual proteins in each family is known. We wanted to see what kind of impact expansion of a family by duplication and divergence has on the host cell. Different paths of divergence would be expected to create the differences one sees in the taxa today. As to what kinds of proteins to examine, we chose to look at enzymes even though they form smaller data sets than those for transport and regulation proteins. Our goal was not to reconstruct evolutionary events over time, but to look at the power of duplication to affect the identity of the cell in specific biochemical terms. We ask in qualitative terms if the content of a family of enzymes bears a relationship to the biological characteristics of the organisms in which they reside.

A companion study to this one from our laboratory, used MrBayes methodology to develop unrooted trees of the enzymes of this study [9]. These data show that the enzyme trees do not correspond to trees of the organisms, nor would we expect them to. Protein family trees are different from phylogenetic trees of organisms. The selection factors that operate on enzymes such as availability and concentration of cofactors, energy supply (e.g. ATP, NADH), interactions within metabolic pathways, response to regulatory chains, tolerance to inhibitors, to ion concentrations, the breadth of substrate accommodation, and so on and so on, need not connect quantitatively with the factors that affect phylogeny of the organism as a whole.

**Figure 1:**
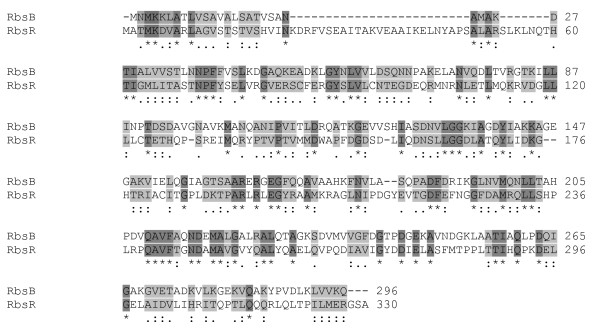
**Alignment of the *E. coli *ribose transport protein RbsB and the ribose repressor RbsR**. The protein sequences were aligned with ClustalW 2.0.11. Identical residues are highlighted in dark grey while conserved and semi-conserved residues are highlighted in light grey.

There have been few studies confined to enzymes as factors in molecular evolution. Jensen in 1976 pointed out the importance of "recruitment" of new enzymes in evolution by gene duplication followed by changes in the specificity of the new copies so as to take on a related, but new role [10]. Some relationships of enzymes within a pathway could be understood in these terms. Another mechanism is duplication and modification of one copy by addition of another domain. An example of such a relationship is the pair of genes in *Escherichia coli *for the ribose repressor (RbsR) and the periplasmic protein for ribose transport (RbsB). These proteins share the sequence spanning the periplasmic binding protein (PBP) domain (PF00352) but differ in the acquisition of a DNA-binding domain by RbsR. An alignment of RbsR and RbsB is shown in Figure 1. While both proteins have maintained their ability to bind ribose using the PBP domain, RbsR has gained the ability to bind DNA and regulate transcription while the RbsB has been modified to allow for export to the periplasmic space and for interaction with the membrane components of the ABC type transporter.

Different from the rbs story, there are families of sequence similar enzymes that use the same reaction mechanisms but vary in substrate specificity. An example is the family of aminotransferases Class III. However, perhaps even more interesting, there are other families of sequence-similar enzymes that catalyze related but different reactions. Such mechanistically diverse collections are called superfamilies of enzymes. Several enzyme superfamilies, isolated from many biological sources, have been studied carefully from a structural and biochemical point of view. These include the enolase, Nudix, amidohydrolase, crotonase and haloacid dehalogenase superfamilies (reviewed in [11]). We have focused on identifying the members of a superfamily within one organism, a group of enzymes that could have arisen by duplication and divergence. We ask whether the members of the family are of a kind that would contribute to the metabolic identity of the organism.

One such superfamily is the Short Chain Dehydrogenase-Reductase (SDR) family. Similarities among certain dehydrogenases from *Streptomyces *spp., *Drosophila melanogaster *and several mammals, led to the identification of a type of dehydrogenase given the name SDR [12]. All reactions catalyzed by members of this superfamily require the cofactor NAD(P)/H and all members possess the Rossman fold. As more and more members of this superfamily were identified, the family was found also to include epimerases, dehydratases and isomerases [13]. It is variations on a theme of reaction chemistry that ties members of the superfamily together. This is different from earlier ideas on evolution of enzymes where a single enzyme would change by modifying substrate affinities, not by varying the reaction.

In the context of evolution, one can ask what kinds of biochemical properties have been conferred on a single organism by this process. To answer the question we decided to gather the members of the SDR family in *E*. coli, and then expand the study to other sequence-related enzyme families, not only from *E. coli *but from other bacteria as well.

## Results and Discussion

To find out how many members of the SDR family are present in *E. coli *K-12 MG1655, henceforth *E. coli*, we assembled enzymes identified with an EC number 1.1.1.x. Among these are enzymes with the structural and sequence characteristics of the SDR superfamily. Initially we used the AllAllDb program of the Darwin system [14] (after first separating independent, fused proteins into their components) to collect all sequence related *E. coli *enzymes from this group. Parameters of the initial pair-wise similarity search were set as requiring a Pam value of at least 200, an alignment of 83 residues and an involvement of at least 50% of the length of the smaller protein of any sequence-similar pair. Related enzymes were assembled by transitive relationship. To extend membership in the groups to include proteins whose sequence may have diverged further, we submitted all members to PSI-BLAST analysis [15].

*E. coli *has 15 members of the SDR family whose substrates and reactions are known (Table 1). We found that the entire superfamily could be subdivided based their sequence similarity into two separate groups. One of these groups contained all the dehydrogenase/reductases, the other all the epimerase/dehydratases. Although the reactions of the second group are not oxidative the apparent anomaly is explained by their reaction mechanisms. For SDR enzymes, reactions of epimerization, dehydration or isomerization are promoted with an oxidation-reduction type of chemistry that promotes both loss and gain of a proton so as to change the placement of the moieties of the substrate or to promote dehydration. Both types of reactions are facilitated by a Ser-Tyr-Lys catalytic triad whose spatial configuration and charge distribution is affected by the binding of each substrate [16].

**Table 1:** List of *E. coli *SDR related enzymes and metabolic pathways.

Gene	Gene Product	Pathway	Phenotype
*fabG*	3-oxoacyl- [acyl-carrier-protein] reductase	fatty acid biosynthesis	synthesis of essential metabolites
*fabI*	enoyl- [acyl-carrier-protein] reductase (NADH)	fatty acid biosynthesis	synthesis of essential metabolites
*hcaB*	2,3-dihydroxy-2,3-dihydrophenylpropionate dehydrogenase	3 phenylpropionate degradation	utilization of aromatic compounds
*srlD*	glucitol (sorbitol)-6-phosphate dehydrogenase	sorbitol (glucitol) degradation	utilization of a sugar alcohol
*idnO*	5-keto-D-gluconate 5-reductase	L-idonate degradation	utilization of a sugar
kduD*	2-deoxy-D-gluconate 3-dehydrogenase	pentose and glucuronate interconversions	interconversion of 5- and 6-carbon carbohydrates
*hdhA*	NAD-dependent 7alpha-hydroxysteroid dehydrogenase	degradation of human bile acids	decomposition of intestinal bile acids
*entA*	2,3-dihydro-2,3-dihydroxybenzoate dehydrogenase	enterochelin/enterobactin biosynthesis	binds and solubilizes iron in enteric bacteria
*rfbD*	dTDP-6-deoxy-L-mannose-dehydrogenase	dTDP-L-rhamnose biosynthesis	biosynthesis of enterobacterial common antigen

*rfbB*	dTDP-glucose 4,6 dehydratase	dTDP-L-rhamnose biosynthesis	biosynthesis of enterobacterial common antigen
*rffG*	dTDP-glucose 4,6-dehydratase 2	dTDP-L-rhamnose biosynthesis	biosynthesis of entobacterial common antigen
*galE*	UDP-galactose-4-epimerase	UDP-galactose biosynthesis	metabolism of galactose; biosynthesis of colanic acid
*fcl*	GDP 4 keto 6 deoxymannose epimerase, dehydrogenase	GDP-L-fucose biosynthesis	biosynthesis of colanic acid
*gmd*	GDP-D-mannose dehydratase	GDP-L-fucose biosynthesis	biosynthesis of colanic acid
*rfaD*	ADP-L-glycero-D-mannoheptose-6-epimerase	ADP-L-glycero-D-mannoheptose biosynthesis	biosynthesis of Lipid A

* predicted activity

The grey line separates the dehydrogenases/reductases (top) from the epimerases/dehydratases (bottom).

**Figure 2:**
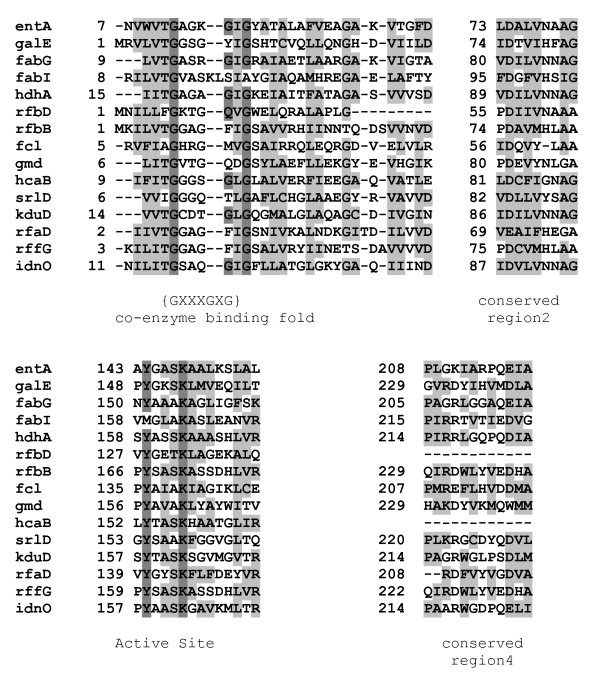
**Alignment of *E. coli *SDR family members**. The enzymes of the family members are listed in Table 1. Four conserved regions of the proteins are shown. The protein sequences were aligned with ClustalW 2.0.11. Identical residues are highlighted in dark grey while conserved and semi-conserved residues are highlighted in light grey.

Examination of the sequence alignments of the *E. coli *SDR enzymes revealed four regions that aligned for all members of the extended family, the substrate binding site, the NAD(P)/H-binding Rossman fold and two sites of unknown function, likely to be important for folding (Fig. 2). Each of the conserved sequences occurs in approximately the same region within each protein. Small changes in the residues in conserved regions have large effects on the affinity for particular substrates and on the specific reaction that is catalyzed.

Table 1 shows the separation into two types of crotonases and the variety of pathways and resulting phenotypes served by the SDR superfamily. Some pathways are used by many organisms, such as fatty acid synthesis, but many products and processes are characteristic of the enteric organisms only, such as bile acid emulsification, biosynthesis of colanic acid, lipid A, enterobactin and enterobacterial common antigen. It appears that the process of duplication and divergence has contributed to the metabolic characteristics of a unique phylogenetic group of bacteria.

One can ask how broad the phenomenon of families is among *E. coli *enzymes. Even before the sequence of the *E. coli *genome was completed, the existence of families of related sequence within its genome was observed [17,18]. Such sequence-related families are viewed as paralogous families that arose by duplication of genes within the genome of the organism itself or in that of an ancestor, although as previously mentioned some members of these families could have been introduced by lateral gene transfer. After completion of the full genomic sequence of *E. coli *[19], the complete set of paralogous families in relation to the whole genome could be determined. Pair-wise related sequences from the entire genome were assembled, using the criteria of similarity as having Pam values below 200 and alignments of at least 83 residues. By requiring an alignment of 83 amino acids or more we seek to avoid grouping sequences by small common domains or motifs, such as DNA binding domains, instead we detect protein level duplications. For example in the RbsR/RbsD case, the 45 amino acid DNA-binding domain (PF00356) is present in 14 additional *E. coli *transcriptional regulators. Since the main components of these proteins, the ligand-binding domains, not are related to RbsR we do not consider them paralogs. Our groups ranged in size from 92 members in the largest group down to the smallest size, simple pairs. Over half of the *E. coli *proteins resided in these sequence-related groups [20-22].

The existence of families of sequence-similar proteins making up a large fraction of the genomic content supports the proposal that duplication followed by divergence is an important mechanism of molecular evolution. The largest groups in the *E. coli *genome were those of related transport proteins, regulatory proteins, and redox (i.e. iron-sulfur) subunits of enzyme complexes. Groups of sequence similar enzymes were smaller, had fewer members, than the groups of transporters and regulators. However, we concentrated on the class of enzymes because studying families of enzymes has the advantage of being able to draw on the detailed knowledge in the extensive biochemical literature concerning their properties, prosthetic groups, the mechanisms of the reactions they catalyze and pathways they belong to. One is in a position to link genetic information with biochemical information and thus with phenotypes of the organism. Examining the members of enzyme families of *E. coli *allowed a view at the molecular level of what kind of creation of function occurred as a consequence of presumed duplication and divergence.

Another superfamily that is structurally and mechanistically related but catalyzes diverse reactions is the crotonase family. The family was originally characterized by similarities in three-dimensional structure of four enzymes derived from different sources. Although structurally related, sequence related and mechanistically related, their biochemistry showed that they catalyzed four different reactions [23]. Subsequent investigation has shown that the crotonase enzymes are related in sequence, though often distantly, and catalyze a broad range of reactions i.e. dehalogenation, hydration/dehydration, decarboxylation, formation/cleavage of carbon-carbon bonds and hydrolysis of thioesters [24].

**Figure 3:**
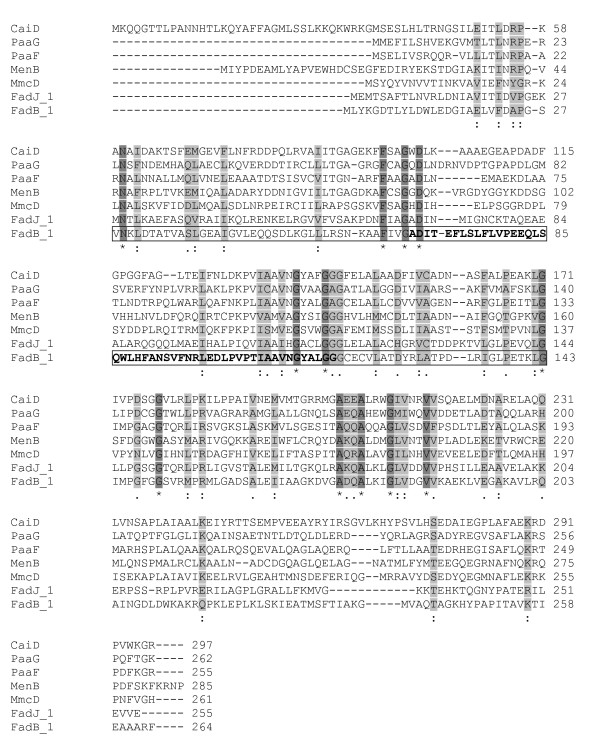
**Alignment of *E. coli *crotonase family members**. Protein family membership was determined as proteins having sequence similarity of 200 Pam units or less over at least 50% of their length. Members of the E. coli crotonase family are listed in Table 3. The protein sequences were aligned with ClustalW 2.0.11. Identical residues are highlighted in dark grey while conserved and semi-conserved residues are highlighted in light grey. Residues forming the FadB oxanion hole used to stabilize reaction intermediates are shown in bold-face. The FadB reaction center is outlined.

To look at crotonases in an evolutionary context, one can ask if they could have arisen by duplication and divergence. To approach this question, one could enumerate all crotonases in one organism. Starting with a crotonase in *E. coli*, encoded in the N-terminal portion of FadB (here designated FadB_1) with demonstrable structural similarity at the active site to the rat liver crotonase, we assembled the group of sequence-similar enzymes in *E. coli *as before by the Darwin AllAllDb program. Figure 3 presents the alignment of residues at the active site for the *E.coli *crotonase family. The greatest amino acid conservation is seen for the residues involved in acyl-CoA-binding and the catalytic site. There is a CoA-binding site and an expandable acyl-binding pocket as well as an oxyanion hole for binding the thioester C = O bond, crucial to the reaction catalyzed by members of this superfamily [23,25]. Variations in residues at critical positions in the active sites dictate which of the related reactions occurs. Again, as for the SDR family, one can visualize that the broad family of crotonases, spanning several kinds of reactions, could have arisen by gene duplication and divergence early in evolutionary time.

By assembling the crotonase family members in a few organisms, one expects that some individual enzymes will be present in all the organisms as they are virtually universal. However other members of the crotonase family are expected to differ from one organism to another. We expect that bacteria in separate lineages would have some enzymes that catalyze different reactions. Differentiation of bacteria as they evolved along different lineages is expected to be partly as a consequence of generating different enzyme family members in the course of the divergence process. Other molecular evolution events are occurring at the same time as the duplication and divergence, such as lateral transfers and gene loss. To focus on gene duplication we decided to look at families of enzymes in a set of both similar and distant bacteria.

We asked whether members of three enzyme families are the same in the bacteria examined or whether there are differences dictated by separate evolutionary histories and separate selective pressures. Three enzyme families were compared in four bacteria. The families chosen for comparison were the crotonases, pyridoxal phosphate-requiring aminotransferases Class III, and thiamin diphosphate-requiring decarboxylases. The four bacteria are *E. coli*, *Salmonella enterica *subsp. *enterica *serovar Typhimurium LT2 (henceforth *S. enterica*), the distant γ-proteobacterium *Pseudomonas aeruginosa *PAO1 and the gram positive bacterium *Bacillus subtilis *subsp. *subtilis *strain 168 (henceforth "*B. subtilis)*.

The families of enzymes were assembled for the three organisms using the same methods as for *E. coli*. Table 2, 3, and 4 list members of the aminotransferase-, decarboxylase-, and crotonase superfamilies, respectively. Known enzymes and strongly predicted enzymes present in each of the four bacteria are shown as well as the number of proteins currently of unknown function.

**Table 2:** Class III Aminotransferase superfamily members.

organisms	se^a^	ec^b^	bs^c^	pa^d^	Enzyme
known function					
	*bioA*	*bioA*	*bioA*	*bioA*^*p*^	adenosylmethionine-8-amino-7-oxononanoate aminotransferase
	*gabT*	*gabT*	*gabT*	*gabT*	4-Aminobutyrate aminotransferase
	*hemL*	*hemL*	*hemL*	*hemL*	glutamate-1-semialdehyde aminotransferase
	*argD*	*argD*	--	--	acetylornithine/succinyl-diaminopimelate aminotransferase
	*astC*	*astC*	--	--	succinylornithine aminotransferase, catabolic
	--	--	*argD*	--	acetylornithine aminotransferase, biosynthetic
	*patA*	*patA*	--	--	putrescine 2-oxoglutarate aminotransferse
	--	*puuE*	--	--	4-Aminobutyrate aminotransferase (putrescine pathway)
	--	--	*gsaB*^*p*^	--	glutamic-1-semialdehyde aminotransferase
	--	--	*rocD*	--	ornithine aminotransferase
	--	--	--	*pvdH*	diaminobutyrate aminotransferase
	--	--	--	*spuC*	putrescine:pyruvate aminotransferase
	--	--	--	*aruC*	succinylornithine aminotransferase, catabolic
				PA5313	4-Aminobutyrate aminotransferase
				*oapT*^p^	β-Alanine:pyruvate aminotransferase

unknown function					
# orfs	--	--	2	5	

family size	6	7	8	13	

^a^*Salmonella enterica *subsp Typhimurium LT2

^b^*Escherichia coli *K-12 MG1655

^c^*Bacillus subtilis *subsp. *subtilis *strain 168

^d^*Pseudomonas aeruginosa *PA01

^p ^strongly predicted to encode the listed enzyme

**Table 3:** Thiamine diphosphate decarboxylase superfamily members.

organisms	se^a^	ec^b^	bs^c^	pa^d^	Enzyme
known function					
	*poxB*	*poxB*	*ydaP*^p^	*poxB*^p^	pyruvate oxidase
	*menD*	*menD*	*menD*	--	2-oxoglutarate decaboxylase, SHCHC synthase
	*gcl*	*gcl*	--	*gcl*^p^	tartronate-semialdehyde synthase
	*ilvB*	*ilvB*	*ilvB*	--	acetolactate synthase I, large subunit
	*ilvI*	*ilvI*	--	*ilvI*	acetolactate synthase III, large subunit
	*ilvG*	ilvG*	-	---	acetolactate synthase II, large subunit
	--	*oxc*^p^	--	--	oxalyl-CoA decarboxylase
	--	--	--	*mdlC*	benzoylformate decarboxylase
	--	--	--	*aruI*	2-ketoarginine decarboxylase
	--	--	*iolD*	--	3D-(3,5/4)-trihydroxycyclohexane-1,2-dione hydrolase
	--	--	*alsS*	--	acetolactate synthase, catabolic

unknown function					
# orfs	1	--	--	5	

family size	7	7	5	10	

^a^*Salmonella enterica *subsp Typhimurium LT2

^b^*Escherichia coli *K-12 MG1655

^c^*Bacillus subtilis subsp. subtilis *strain 168

^d^*Pseudomonas aeruginosa *PA01

^p ^strongly predicted to encode the listed enzyme

* contains internal frameshift mutation

**Table 4:** Crotonase superfamily members.

Organisms	se^a^	ec^b^	bs^c^	pa^d^	enzyme
known function					
	*menB*	*menB*	*menB*	--	napthoate synthase
	*fadB_1*^e^	*fadB_1*	*fadB*	*fadB_1*	fatty acid oxidation complex subunit
	*caiD*	*caiD*	--	--	carnityl-CoA dehydratase
	*fadJ_1*	*fadJ_1*	--	--	fatty acid oxidation complex subunit
	--	*paaF*	--	--	enoyl-CoA enzyme
	--	*paaG*	--	--	enoyl-CoA enzyme
	--	*mmcD*	--	--	methylmalonyl-CoA decarboxylase
	--	--	*yngF*	--	hydroxybutyrlylmethyl-CoA dehydratase/methylcrotonyl-CoA carboxylase
	--	--	*pksH*	--	dehydratase
	--	--	*pksI*	--	decarboxylases
	--	--	--	*liuC*	methylglutaconyl-CoA hydratase
	--	--	--	*atuE*	Isohexenyl-glutaconyl-CoA hydratase

unknown function (orfs)					
	0	0	2	14	

family size	4	7	7	17	

^a^*Salmonella enterica *subsp Typhimurium LT2

^b^*Escherichia coli *K-12 MG1655

^c^*Bacillus subtilis *subsp. *subtilis *strain 168

^d^*Pseudomonas aeruginosa *PA01

^e ^"_1" designates the N-terminal portion of the gene

We note that some of the enzymes are present in all four bacteria, suggesting they are integral parts of core metabolic functions. This is supported by the pathways they participate in; biotin synthesis and porphyrin synthesis (BioA and HemL), aminobutyrate utilization (GabT), pyruvate oxidation (PoxB/YdaP), and fatty acid oxidation (FadB). One supposes such commonly held important functions are conserved in many bacteria in many taxa.

Other enzymes differ in their distribution (presence or absence) among the four organisms. This is presumably a result of different evolutionary histories in different lineages during the divergence processes, leading to establishment of bacterial taxa with biochemical and metabolic differences. For example the MenD decarboxylase and MenB crotonase used for menaquinone biosynthesis are absent from *P. aeruginosa *and present in the other three organisms. This distribution is reflective of the Pseudomonads using only ubiquinone, and not both ubiquinone and menaquinone, as electron carriers for respiration. Gcl, tartronate-semialdehyde synthase of glyoxalate utilization, is present in three bacteria, and not in *B. subtilis*. Degradation of glyxolate in *B. subtilis *has been shown to occur by a different pathway from the other three organisms. In the two enteric organisms, their particular paths of metabolizing putrescine and carnitine are reflected in the presence of putrescine aminotransferase (PatA) and carnityl-CoA dehydratase (CaiD) in both *E. coli *and *S. enterica*.

Several of the aminotransferases are involved in arginine metabolism, and the occurrences of these enzymes also vary among the organisms. *E. coli *and its close relative *S. enterica *both have ArgD and AstC for biosynthesis and degradation of arginine, respectively. AruC is used by *P. aeruginosa *for both arginine synthesis and degradation. While in *B. subtilis*, ArgD is used for arginine synthesis and RocD, another member of the aminotransferase family, is used to degrade arginine by a different pathway. We observe that the two more closely related enteric organisms have a higher similarity in their aminotransferase content.

Some of the protein family members represent isozymes, sequence similar enzymes that catalyze the same reaction but with definable differences such as substrate breadth, feedback inhibition, binding constants, reaction rates and the like. Based on the common nature of the isozymes, we suppose they have arisen by gene duplication and slight divergence. Examples of isozymes are the trio of acetolactate synthases; IlvB, IlvI and IlvG, found in *E. coli *and *S. enterica*. These isozymes function in the isoleucine and valine biosynthesis pathway, each responding to distinct feed back. One copy, IlvG, is mutated and inactive in *E. coli*, rendering *E. coli *valine sensitive. This phenotype is used in identification protocols to distinguish *E. coli *and *S. enterica*. A second type of acetolactate synthase (AlsS) is also present in *B. subtilis*, but this enzyme is used exclusively for catabolism and not synthesis of isoleucine and valine.

*E. coli *and *S. enterica *have another set of isozymes, FadB and FadJ. Both enzymes are used for fatty acid oxidation, but FadB is used under aerobic conditions and FadJ is used under anaerobic conditions. Other isozymes are GabT and PuuE in *E. coli*, GsaB and HemL in *B. subtilis*. Isozymes are often specific to pathways, such as PuuE, which is specific to putrescine utilization. One supposes that simply by small changes in duplicate genes, pathway content and biochemical capability of an organism can expand.

In addition there are protein family members that are unique to only one of the four organisms and absent in the other three. These enzymes often confer metabolic properties unique to their host. An example is oxalyl-CoA decarboxylase (Oxc) that is present *E. coli *where it is believed to confer oxalate degrading capabilities. As is the case for any of the enzymes present in one organism, not the others, the gene could have been acquired by lateral transmission [26]. However when an enzyme like oxalyl-CoA decarboxylase, is found in many bacteria, it is at least as possible that it arose by gene duplication and divergence. Other organism specific enzymes, in this case *B. subtilis*, include the IolD for myo-inositol degradation and the crotonases PksH and PksI used for polyketide synthesis. Polyketides are a group of secondary products peculiar to the Bacilli. Other unique *B. subtilis *enzymes AlsS, GsaB and RocD have been mentioned above. It seems evident that formation of different enzymes by unique divergence events, add up to creation of taxa with different metabolic characteristics.

*P. aeruginosa *has the largest number of unique, or organism specific, enzymes in our dataset. This is shown for all three enzyme families (Tables 2, 3, 4). These *Pseudomonas *specific enzymes include synthesis of the siderophore pyoverdine (PvdH), and utilization of mandelate (MdlC), leucine and isovalerate (LiuC) and acyclic terpenes (AtuE). Other predicted family members include two aminotransferases: PA5313, evidently an isozyme for 4-aminobutyrate, and OapT, likely a beta-alanine:pyruvate enzyme. Each of these enzymes contributes to the distinct metabolic character of *P. aeruginosa *as a pseudomonad. In addition there are 5 aminotransferases, 5 decraboxylases and 14 crotonases whose functions remain unknown in *P. aeruginosa*. Our phylogenetic analysis [9] suggests that these are unique enzymes representing additional functions yet to be discovered. Combining genes of known and unknown function for the three families, the number of unique *P. aeruginosa *genes (33) far surpasses that of *B. subtilis *(12), *E. coli *(2) and *S. enterica *(1). The large number of *Pseudomonas *specific enzymes detected is in agreement with the well-documented metabolic versatility of this group [27,28].

These examples of differences among enzyme families in four organisms suggest that the distinct events of divergence in genes of protein families over time have generated taxa of bacteria that are distinguished in part by their metabolic differences. Bacteria that are closely related have fewer differences in these families. For all three enzyme families we noted that the two most closely related organisms, *E. coli *and *S. enterica*, contain the most similar complement of enzymes. Larger differences in both number of dissimilar enzymes and enzyme functions were seen when comparing either *B. subtilis *or *P. aeruginosa *to any of the other three.

Overall, our protein family analysis includes several examples of how the functional and metabolic diversity of today's organisms is reflected in a history of duplicated and diverged gene copies in their genome sequences. In some instances the gene copies are the same in all the bacteria. These are enzymes for universal functions. Some of the gene copies did not undergo much divergence and resulted in isozymes catalyzing the same reactions but with different properties. Such enzymes usually contribute to phenotypic differences, for instance by changes in substrate specificity or regulation. Still other gene copies were not found in other bacteria. These were functions characteristic of the phenotype of the particular organism. We do not suggest that duplication of genes was the only source of diversity in these organisms. In addition there lateral transfer could have introduced a new function and also gene losses would have changed the composition of protein families. Some analyses suggest that lateral gene transfer has played a large role in assembling gene families [29]. However one needs to take into account the lack of congruence between organism trees and gene trees, the latter being affected by different selective pressures on individual enzymes (such as gene family composition, cofactor/substrate availablility) compared to those affecting the organism as a whole. Lawrence and Hendrickson [30] have discussed in a thoughtful way the difficulties in distinguishing horizontal transmission from duplication of existing genes. We have therefore not attempted to identify laterally transferred genes in our enzyme families. While possibly there we do not expect them to predominate. In summary, it is a combination of all these genetic changes (duplications, divergence, loss and acquisitions) in ancestors of contemporary organisms that has generated the characteristic phenotypes of today's organisms.

## Conclusion

By assembling selected superfamilies of enzymes of sequence and structural similarity in four different bacteria whose entire genomes have been sequenced, we suggest that members of the families arose in the course of evolution at least in large part, by duplication followed by divergence. We observed that differences in the enzyme families, both in functions and numbers of homologs, were greater as the organisms were less closely related. Functional differences of family members were reflective of metabolic diversity of the host genome. Events such as gene loss and gain also must have made changes to enzyme family rosters over time, but we suggest that the outline of the duplication and divergence process remains visible in the contemporary paralogous groups of sequence-related superfamilies.

### The future

The examples here of enzyme families that could have arisen by duplication and divergence are only representative of a large number of such sets of sequence related proteins in all organisms. Continuing to assemble and analyze such families will undoubtedly bring more understanding to the mechanisms of their origins and the relationships of enzymes and pathways to the life style of each organism. Including proteins other than enzymes will paint a more detailed and well-rounded picture of the span and significance of gene duplication as a mechanism of molecular evolution.

All other avenues of molecular evolution in terms of protein sequences will continue to be pursued using the grist provided by the ever-increasing collection of complete genome sequences. A different avenue of phenotypic change that goes beyond presence and absence of protein sequences is the immense arena of epigenetics. The complexities of many systems in regulation of gene expression have the potential of bringing about evolutionary changes that would not be visible in the sequences of the proteins being regulated. Small genetic changes, in small regulatory RNAs, for instance, affecting complex multi-gene regulatory systems may give rise to far-reaching phenotypic changes [31-34]. It seems likely that future research on physiological functions affected by epigenetic differences will bring new insights into the processes of evolution. Incorporating data in a systems approach will be a way to include regulation as an important factor affecting molecular evolution [31].

## Methods

Pairwise sequence alignments and scores were generated using the AllAllDb program of Darwin (Data Analysis and Retrieval With Indexed Nucleotide/peptide sequence package), version 2.0, developed at the ETHZ in Zurich [14]. Maximum likelihood alignments are generated with an initial global alignment by dynamic programming followed by dynamic local alignments. A single scoring matrix is used for these steps. After the initial alignment, the scoring matrix is adjusted to fit the approximate distance between each protein pair to produce the minimum Pam value. Pam units are defined as the numbers of point mutations per 100 residues [35,36].

### Protein sequences

UniProtKB Accession numbers of the proteins used in the analysis included; Ribose Regulator and Transport proteins (P0ACQ0, P02925), Short Chain Dehydrogenase-Reductase family (P15047, P0AEK2, P0AEK4, P0AET8, P77646, P05707, P37769, P0A9P9, P37760, P32055, P09147, P67910, P37759, P0AC88, P27830), Class III Aminotransferase family (P36839, P94427, P53555, P71084, P30949, P33189, O34662, P38021, P22256, P50457, P12995, P23893, P48247, P18335, P77581, O30508, Q9I606, Q9I6 M4, Q9I693, Q9I700, Q9I6J2, Q9I6R7, Q9HV04, Q9HTP1, Q9HWU0, Q9HT50, P48247, Q9I168, P12677, Q82 MM1, P21267, P40732, Q8ZPV2, Q8ZLX7), Thiamine Diphosphate Decarboxylase family (P96591, P37251, P23970, Q04789, P42415, P07003, P0AEP7, P08142, P00892, P00893, P17109, P0AFI0, Q9HTQ7, Q9I3L0, Q9HVA0, Q9I3S7, Q9HUI8, Q9HUR2, Q9I280, Q9I207, Q9HYA3, Q9HWK1, Q8ZQF0, Q8ZNE8, Q8ZR85, Q8ZL15, P40811, Q9L6T2, Q93IM7), Crotonase family (P23966, P40805, P40802, O07533, O34893, P94549, O32178, P0ABU0, P76082, P21177, P77399, P31551, P52045, P77467, P76082, Q9I498, Q9I002, Q9I393, Q9HY35, Q9HZJ2, Q9I300, Q9HZV7, Q9I298, Q9I5I5, Q9HW71, Q9HUI5, Q9I2S4, Q9I5I4, Q9I4V3, Q9I2Y9, Q9I076, Q9HYH9, Q9L6L5, Q8ZNA7, Q82RX5, Q7CQ56).

Sequence pairs were collected that had alignment lengths of at least 83 amino acids, distances of 200 Pam units or less, and aligned over at least 50% of the length of the query sequence. Multimodular proteins were identified and separated into modules of independent functions. We chose the length requirement of 83 residues as it improves the significance of the sequence alignments for the more distantly related protein pairs [37,38]. The requirement for at least 83 residues also avoids a class of commonly occurring protein domains smaller than 83 residues that appear widely in many otherwise unrelated proteins (such as small binding sites for a type of substrate or cofactor or regulator).

To extend to a lower level of similarity, the PSI-BLAST program [38] was used to collect successively sets of related proteins. SEG filtering was used and the search limited to no more than 5 rounds. This allows for more divergent set of sequences to be grouped. Proteins were removed from a result if they had poor matches to only one or two group members. Families were formed by transitive clustering.

Sequence alignments were generated with the ClustalW program [39].

For the proteins having weak sequence similarities, validity of including these proteins in a family was established using structural features. We undertook to thread sequences of weakly similar proteins sequences on the structure of a representative bacterial protein template. To choose a template representing each of the three functional groups, the whole-protein sequence of the most closely related bacterial members of each group were used as queries to search the PDB database [40] using the *blastp *program of the BLAST package [41].

The closest structural neighbor (template) in bacteria was chosen based on the highest similarity scores. The selected templates were structures of the rat crotonase (PDB ID: 2DUB), *Pseudomonas fragi *FadB (PDB ID: 1WDM), *E. coli *BioA (PDB ID: 1DTY, *Lactobacillus plantarum *Pox (PDB ID: 1POW). Structural models were generated using the DeepView - Swiss-PdbViewer application (version 3.7) and the MagicFit tool [42]. Pairwise alignments between the primary amino acid sequence of each target and the respective structural template were performed using DeepView. To get the optimum structural alignment, the Magic Fit and Iterative Magic Fit functions were used. This super-imposition generated the structural coordinates for the modeled proteins.

## List of Abbreviations

SDR: short chain dehydrogenase-reductase; NAD(P)H: nicotinamide adenine nucleotide (phosphorylated) reduced; Orfs: open reading frames; CoA: coenzyme A; Pam: point accepted mutations.

## Competing interests

The authors declare that they have no competing interests.

## Authors' contributions

Original conception by MR, MHS and MR wrote the paper, most data collection and analysis was done by MHS, also by TJC for crotonases, ARWK for SDR family, and MR for *Salmonella enterica *serovar Typhimurium LT2.

## Reviewers' comments

Referee 1:

Pierre Pontarotti

Directeur de Recherche CNRS

Marseilles, France

### Reviewer comments

I carefully read your article with great interest. Unfortunately, I do not see any new information in your article. Indeed, gene duplication related to functional evolution has been highly described in the literature as well as the link with physiology.

Maybe I miss something: if this is the case, I suggest that you should better explain the originality of your work to the reader and you also could provide a comparative description with the already published articles.

Despite this comment, the analyze is straightforward and carefully carried out.

### Authors' response

We appreciate your prompt reading of our paper. We can see that we have not done a good job of explaining how our study differs from others. Many studies of gene duplication gather total data on sizes of paralogous families in organisms, analyze numbers and rates of mutation etc., as a mathematical model, but do not bring into the picture the difference in functions developed by some of the duplications. We have purposely undertaken to examine closely just a few paralogous families where in most cases the enzymes made by the genes in the families are known. This allows us to see what functions are in common in the chosen microorganisms and what functions have arisen presumably by mutation that are specific to one organism or to closely related organisms, but not to others. In other words, since we know what these gene products do, what pathways they participate in, we can learn something about how organisms became differentiated and unique from one another in biochemical terms.

We will be making this point much more clearly in the manuscript now, thanks to your comments. If you know of other studies along these lines that we should be aware of, it would be a kindness to direct us to them.

Referee 2:

Iyer Aravind

NCBI, NIH

Bethesda, MD

### Reviewer's comments

"These proteins share many sequence similarities except that the repressor has a DNA-binding sequence at the N-terminal end, but the transport protein does not."

-This sentence should be modified to simply reflect the fact that the proteins share a PBP domain and that the transcription regulator has acquired a DNA-binding domain.

"Pair-wise related sequences from the entire genome were assembled, using the criteria of similarity as having Pam values below 200 and alignments of at least 83 residues. The groups ranged in size from 92 members in the largest group down to the smallest size, simple pairs."

-This is an underestimate of the actual paralogy situation in the genome. A disclaimer to this effect would be appropriate, indicating that the above method provides an approximate estimate of the cluster sizes of paralogs in the proteome. It might also be proper to differentiate between the paralogy of domains and whole proteins like the RbsR/RbsB example discussed above.

"...(CaiD) in both E. coli and Typhimurium."

-Better to spell out the whole name Salmonella typhimurium and thereafter use S.typhimurium

"P. aeruginosa has a large number of such single organism occurring enzymes"

-The sentence is highly agglutinative, could modified to express the point better. Secondly a more quantitative estimate of the "large number" would be useful. A comparison relative another organism could also be of value.

"...we suggest that members of the families arose in the course of evolution at least in large part, by duplication followed by divergence."

-This statement is entirely true, but it seems to be a bit of a platitude in this context because the introduction itself starts of stating the role of duplication in diversification of protein families. Certainly the protein families have emerged through this process. But what does the "large part" mean? Does it imply that a part of the family did not arise by this process? Or are the authors trying to say within a genome in large part the process was one of duplication/divergence but a smaller fraction could be lateral transfer.

-This leads to a more general issue regarding the current article. The conclusions would possibly benefit from a more explicit delineation of the relative contributions of lateral gene transfer and lineage-specific expansions of genes (i.e. duplications) in the evolution of families considered here. In terms of physiological adaptation there is ample evidence from hyperthermophiles and photosynthetic organisms that gene transfer between phylogenetically distant lineages is a major contributor to the paralog complement of these organisms and their proteomes in general. This raises the possibility that in the adaptive transition to new niches the acquisition of genes by lateral transfer is a big player.

-Regarding the final discussion on epigenetics: It is known that proteins mediating epigenetic controls are very variably distributed across the bacterial phylogenetic tree. So is it correct to generalize a major role for epigenetics? Probably not -- it might provide some fine-tuning mechanisms but is unlikely to make a fundamental physiological difference for after the more fundamental determinants are directly inferred from the proteome.

### Authors' response

Thank you for helping us improve our manuscript with your many insightful comments and helpful suggestions. We have adopted or addressed these as follows.

The sequence relationships of RbsR/RbsB has been explained as similarity and differences in domain content.

We have explained that the sequence similar groups we generate not are based on similarity of smaller domains or motifs, but rather require larger fractions of the proteins to be aligned, in an attempt to simulate gene duplication. As a result our estimates of paralogy may be considered conservative.

*Salmonella enterica *subsp. *enterica *serovar Typhimurium LT2 is now referred to as *S. enterica *rather than *S. typhimurium *so as to conform to current correct nomenclature.

We have clarified our statement about the large number of single organism occurring enzymes in *P. aeruginosa *and have included specific numbers and comparisons between the organisms analyzed.

On the influence of duplication and divergence versus lateral transfer as well as gene loss on the current protein family compositions, we have opted not to quantify these sources. We feel that our dataset is too small both in the number of enzymes and organisms compared to make such calculations. When selecting our dataset we sought to use experimentally characterized model organisms and families where the members had known metabolic functions. We have modified the discussion section to further state how gene loss and lateral gene transfer influence today's family compositions, but that based on the difficulty in distinguishing horizontally transferred genes from gene duplications and divergence (Lawrence and Hendrickson reference) we opted not to make such estimations for our dataset.

The section on epigenetics has been slightly modified. While the role of epigenetics may not be the major force affecting evolution of protein families and phenotypes of organisms, we do believe it represents an area of potential new insights into how functional diversity arises and is maintained in organisms.

Referee 3:

Arcady Mushegian

Stowers Institute

Kansas City, KA

### Reviewer's comments

The manuscript deals with the fates of duplicated genes in bacterial genomes, focusing on the selected families of the enzymes with related, diverged functions and their sequence homologs. In the last 15 years, there has been a considerable amount of work on the subject, relating to each other such factors as rate of duplication, rate of duplicate retention, rate of sequence divergence between duplicates, subfunctionalization, speciation, etc. Many of the relevant papers from this corpus of work are cited in this manuscript. The manuscript would benefit from engaging with these cited papers in a constructive way, i.e., by trying to apply some of the quantitative estimates obtained by other workers to the cases that are studied here.

More specifically, I would like to see much more definitive statements about the timing of gene duplication within the selected three families vs. splits of the lineages that the authors study. Polytomies or lack of support for deep nodes in the tree may be a real problem in the subset of cases, but the analysis should be attempted anyway, and specific cases when the results lack support should be noted.

#### Abstract

"Sequence related families of genes and proteins" is perhaps a tautology - "families" already means "sequence-related", does it not?

"In Escherichia coli they constitute over half of the genome." - the total length of these genes is indeed likely to be over half of the genome length; but for this statement to be accurate, the length of the non-coding regions needs to be added to the denominator - has this been done? In fact, I suspect that the authors meant "over half of all proteins encoded by the genome"

"Equivalent families from different genera of bacteria are compared." - what does "equivalent" mean - homologous, of same size, or something else?

"They show both similarities and differences to each other." - consider deleting?

"At least some members of gene families will have been acquired by lateral exchange and other former family members will have been lost over time." - is it "will have been", i.e., expected of the data, or "have been", i.e., shown in this work?

"These families seem likely to have arisen during evolution by duplication and divergence where those that were retained are the variants that have led to distinct bacterial physiologies and taxa." - hard to argue with this, and yet: what would the alternative explanation be - purely stochastic expansion and shrinkage of the families?

#### Background

Par. 1 "Darwin formulated the Origin of Species" - either formulated the theory of Origin of Species, or written The Origin of Species perhaps?

Par. 3, last line: "Stepwise" means "relatively large" in context, but perhaps it should be made more explicit (otherwise, may be interpreted as "step by step", i.e., gradual).

Par. 4: the example of recruitment that the authors discuss is apparently recruitment by addition of novel domain. This is one mechanism of acquiring new function, but I am not sure that this is what R. Jensen meant; as far as I know, his thoughts were more along the lines of sequence drift and polyfunctionality.

Par. 5: "Some attempts to quantify the importance of horizontal, or lateral, transmission in the bacterial genome conclude that foreign gene uptake rather than gene duplication has been a large player in assembling a genome [29]." - I do not think that the study by Lerat et al. is an either/or proposition. They show that a large absolute number of detected gene transfers can coexist with the low frequency of such transfers in most gene families, which is in my opinion a profound result. They do not argue that gene duplication is less important than horizontal transfer, nor I think have their results been disproved. I agree with the authors' approach expressed in the rest of this paragraph, so I think an attempt to argue against the role of HGT is a red herring.

Last paragraph in the Introduction: "In the context of evolution, one might ask whether the genes for this expansive superfamily in one organism (not from many organisms) bear similarity to one another in their sequences." The authors already asserted that SDR is a superfamily - or is it a family, as both terms are used seemingly interchangeably in this paragraph? On what basis has this been established? Most likely, it was sequence similarity (I have no evidence that structures were matched directly, and indeed similarity comparison is what the first paragraph of the Results also suggests), in which case why this needs to be investigated again, or what are perhaps more specific questions that need to be addressed?

#### Results and Discussion

par. 4 - consider deleting?

par. 5 "The groups ranged in size from 92 members in the largest group" - please mention that this is from one study with a conservative similarity threshold; the current count for Walker-box ATPases/GTPases seems to be more than 120 members...

par. 7 "sequence and mechanistically related" - replace with "related by sequence and showing similar molecular mechanism"?

par. 8. Is it important to the authors to make sure that they know all members of each family in E.coli? If the answer is yes, is the AllAllDb comparison sufficient, or perhaps better to build an HMM or a PSIBLAST profile of the already known members and scan the proteome again? If the answer is no, why not?

par. 9: "Some of the SDR enzymes and some of the crotonases are almost universally present in organisms in all three domains of life. Thus one pictures the generation of these enzymes as happening early in evolutionary time, distributed vertically to most organisms." - one may wish to build a phylogenetic tree of the family and compare it with the tree of species to see whether there is any direct evidence for or against horizontal transfer - why not?

Ibid. "Some family members will be virtually universal, but others will differ from one organism or taxa to another, contributing to differences in phenotypes in separate lineages." - is this a statement of the fact or a prediction?

par. 10: "members of three enzyme families are the same in other bacteria" - what does 'the same' mean here?

par. 12: "One supposes such commonly held important functions could have arisen by duplication and divergence early in evolutionary time." - why one has to suppose it - can this again be evaluated by comparing species tree and gene tree?

the next paragraphs: interesting differences are discussed, but no specific evolutionary scenarios are proposed viz. the timing of the events. Can one distinguish between 1. the presence of an enzyme in the common ancestor of the lineages under study (ie., more or less in the common bacterial ancestor) with secondary loss in some of the lineages and 2. emergence of a specific family member by duplication in some but not all of the lineages? When a horizontal transfer event is suspected (e.g. "As is the case for any of the enzymes present in one organism, not the others, the gene could have been acquired by lateral transmission [26]. However when the enzyme is one of a family of similar enzymes, it is at least as possible that it arose by gene duplication and divergence."), why not attempt to sort out what was actually going on?

### Authors' response

Thank you for having taken the time to look carefully at the manuscript. In response to your comments, we have done a major rewrite, during which we incorporated all suggestions about language and expression. We have expanded explanations and have tried to make much clearer the basic thrust of the paper.

In the first part of your review you suggest we do quantitative analysis to sort out when duplication occurred, when divergence occurred, plus when gain of genes by lateral transfer and loss of genes occurred. Our data set is much too small to undertake this type of analysis. We have expanded discussion to include this explanation in the revised manuscript.

You ask what alternatives there are to the process of duplication and divergence. We agree that alternatives are stochastic changes, or perhaps horizontal transfer. But mainly we are saying that one mechanism, perhaps the most important force, in creating the different kinds of bacteria today was duplication and divergence.

We have considered the issue of how we could try to quantify the importance of Lateral Gene Transfer in the four enzyme families we deal with, but we see no obvious outliers in our family groups. Members of these families do not deviate from properties of other members, thus if they came from another host source, time has brought about "amelioration", therefore they are not clearly identifiable as horizontally acquired. We agree that the issue is a "red herring" and have minimized discussion of it in our rewrite.

We have clarified that the definition of the SDR family was originally based on similarity of structure of the regions of substrate binding, cofactor binding and reaction site. Sequence similarity followed soon. The referenced papers give this history.

To our knowledge we are alone in having gathered all members of this family and the others in this paper from a single organism, as detected by the methods we describe, Darwin AllAll algorithm and PSI-Blast. These have been known already as paralogous groups. We are emphasizing their likely formation by duplication and divergence.

It is not surprising to find that there are more Walker ATPase/GTPase motifs than there are ATP-binding subunits of transporters because this motif appears in some other proteins such as helicases.

Reviewer suggests we might build phylogenetic trees of these families. This has been done in a prior report from our laboratory, which we referenced. In our extensive revision we give our reasons for not expecting gene trees for enzymes to be the same as RNA trees representing species.

As to the last comment by the referee, the goal of determining the history of each family of enzymes that led to the distribution and characterization seen today. We have explained in the revision that we have too small a data set to do retrospective analysis, building trees of how the enzymes were generated in each bacterium. Trees of these enzyme families as of today have been presented in a previous publication. We are not able to determine with our data set when specific losses occurred, or whether any of the genes were acquired by LGT. In our revision we have tried to explain much more clearly that this is a qualitative, not quantitative study. What we observe is perhaps no more than common sense, but we show how differences in the members of an enzyme family (divergence) are the kinds of differences that make each bacterial genus unique. Divergence of duplicate enzymes generated differences we now use to characterize bacterial genera.
